# Not Always the Herpes You Think: A Case Report of Genital Zoster

**DOI:** 10.7759/cureus.109149

**Published:** 2026-05-18

**Authors:** Diane V Reed

**Affiliations:** 1 Internal Medicine, University of California Los Angeles, Los Angeles, USA

**Keywords:** dermatomal, dermatomal pattern, genital rash, genital zoster, herpes simplex infection, herpes zoster, herpes zoster virus, vesicular rash

## Abstract

Herpes zoster (HZ), caused by reactivation of the varicella-zoster virus (VZV), is a common viral infection typically presenting with characteristic dermatological findings. However, less typical manifestations, such as genital zoster, are frequently overlooked. This is often due to the higher prevalence of herpes simplex virus (HSV) infections in the genital region, which can lead to diagnostic confusion. Clinicians should maintain a high index of suspicion for genital zoster, particularly in patients with dermatomal rashes or those who are immunocompromised. Accurate diagnosis is essential, as misdiagnosing genital zoster as HSV can result in unnecessary psychological distress and prolonged prophylactic antiviral treatment for some patients. Improved clinical awareness and diagnostic precision can lead to better physical and psychological outcomes for patients. This case report highlights a missed diagnosis of genital zoster, ultimately confirmed by polymerase chain reaction (PCR) testing for VZV, and underscores the importance of considering this condition in the differential diagnosis of genital symptoms, especially in patients with relevant risk factors and clinical features suggestive of zoster.

## Introduction

Herpes zoster (HZ), commonly known as shingles, is a viral infection caused by the reactivation of the varicella-zoster virus (VZV). Around 30% of people will experience an episode of HZ decades after a chickenpox infection or exposure [1]. The annual incidence of HZ in the United States is approximately three to four cases per 1,000 persons, with over one million cases annually [2]. The risk increases dramatically with age, reaching approximately 11 cases per 1,000 persons by the ninth decade of life [3]. Importantly, unvaccinated persons who live to 85 years have a 50% risk of developing HZ [2]. Although it is a frequently encountered diagnosis, certain presentations, such as genital zoster, can be easily overlooked. Because genital zoster mimics sexually transmitted infections such as herpes simplex virus (HSV), it may lead to diagnostic confusion. Genital zoster, while less common than other forms involving the cranial and thoracic dermatomes, can cause morbidity if not recognized and treated. Sacral dermatomes (S2-S4), which include the genital region, are involved in up to 2% of HZ cases [4]. Given this typical distribution, involvement of sacral dermatomes and genital presentations is uncommon and may lead to diagnostic uncertainty, as illustrated in the present case. Early recognition is crucial to initiate antiviral therapy promptly, reduce symptom duration, and prevent complications such as postherpetic neuralgia or secondary infection. Approximately one in five patients with HZ develops postherpetic neuralgia, defined as pain persisting for at least 90 days after rash onset [3]. Multiple case series recommend molecular testing to confirm the diagnosis in all cases of genital herpes-like lesions, testing for both HSV and VZV. The sensitivity and specificity of polymerase chain reaction (PCR) for VZV DNA are 95% and 100%, respectively, making it the preferred test [2]. PCR has well-established diagnostic performance, particularly in lesion-based testing, where it demonstrates higher sensitivity than culture in genital herpes and high specificity in mucocutaneous infections, detecting HSV in 59% of genital lesion cases versus 48% by culture [4]. Misdiagnosis can also cause psychological distress due to the sensitive nature of genital symptoms and the social stigma associated with sexually transmitted infections. Genital zoster often occurs in immunocompetent patients without HIV or significant immunosuppression, making the misdiagnosis more likely to cause unnecessary concern about sexual transmission [5]. This case report highlights a missed diagnosis of genital zoster and underscores the importance of considering this condition in patients presenting with genital symptoms, particularly in those without sexually transmitted disease risk factors and exam findings that have zoster features.

## Case presentation

**Figure 1:**
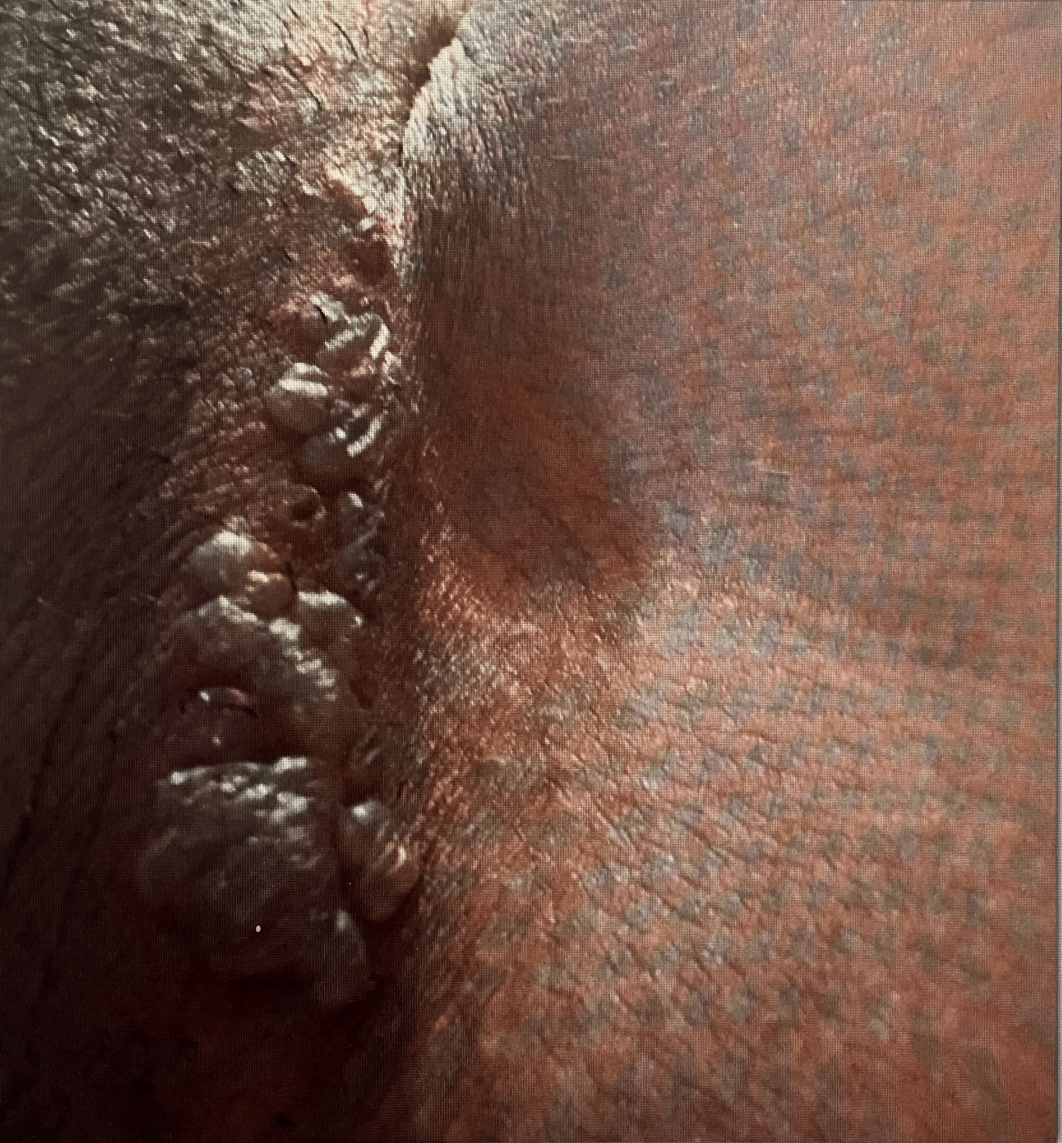
Left-sided vulvar erythematous clustered vesiculopapular lesions

A 29-year-old female patient with no significant past medical history and no history of varicella vaccination presented to the clinic with a one-week history of a vulvar rash. She described the rash as "painful bumps" that were sensitive to touch, accompanied by vulvar swelling (Figure 1).

Notably, the rash was localized to the left side of the vulva, with no similar or characteristic dermatologic lesions observed elsewhere on the body. The patient denied any prodromal systemic symptoms such as fever or malaise. She did report a localized burning-type pain in the affected area prior to rash onset, without associated numbness or tingling. No lymphadenopathy was noted. Additionally, she reported no new vaginal discharge or urinary symptoms, including dysuria. The patient mentioned that the only notable event preceding the rash was shaving the genital area the day before its onset. She had never experienced a similar rash previously and had been in a monogamous relationship with one male partner for several years. The patient also denied any history of sexually transmitted infections (STIs). She reported no recent acute stressors and had no known immunocompromising conditions. Although she did not recall receiving a childhood varicella vaccination, she was otherwise up to date on routine immunizations.

At home, the patient attempted self-care with warm compresses and aloe vera gel, but these measures provided no relief. Due to the persistence of symptoms, she sought care at an urgent care center three days after the rash appeared. During the visit, a left labial lesion was noted. She was tested for HSV types 1 and 2, and a full STI panel (gonorrhea, chlamydia, syphilis, and HIV) was performed. The patient was started on valacyclovir (Valtrex) 1 gram twice daily while awaiting results.

When the HSV PCR returned negative, the patient sought further evaluation from her primary care provider. A repeat examination revealed a linear rash in a vertical distribution involving only the left labium majora, with hyperpigmented, dried vesicles. Given the clinical appearance, a PCR test for VZV was ordered, which returned positive (Table 1). 

**Table 1:** PCR testing HSV: herpes simplex virus; PCR: polymerase chain reaction.

Virology, Direct detection	Result
HSV Type 1 DNA	Not detected
HSV Type 2 DNA	Not detected
Varicella zoster PCR	Detected

Once the VZV PCR result returned positive, the patient’s antiviral therapy was adjusted and valacyclovir was increased to 1 g three times daily. The patient reported that her pain remained manageable and declined the use of analgesics, including neuropathic pain medications. Sitz baths were recommended to assist with symptomatic relief. Follow-up for clinical resolution was arranged via messaging, and an in-person visit was recommended if there was no improvement or complete resolution of the rash. In-person follow-up was not ultimately required given complete resolution of symptoms over the ensuing weeks.

## Discussion

Genital zoster is an uncommon and often overlooked diagnosis. A case series from a sexually transmitted infection clinic in Spain, which included 1,254 patients presenting with genital ulcers or vesicles between 2016 and 2019, found that only 2.8% were diagnosed with genital HZ, compared to over half who were diagnosed with HSV types 1 or 2 using PCR testing [5]. In terms of HZ's typical distribution across dermatomes, a 1990 study reported that only 2% of cases involved the genital region, with most cases originating from the thoracic dermatome [6]. Similarly, a 2018 paper documented the rarity of sacral dermatome involvement, noting it accounted for just 2% of all zoster infections [7]. Due to its infrequent occurrence, genital zoster is not always top of mind for clinicians. For instance, in a study conducted in Australia, 6,210 patients with genital lesions were tested for HSV-1, HSV-2, and VZV using a single sample. Of these, 2,225 had detectable viruses on PCR testing, with over 97% identified as HSV-1 or HSV-2 and 3% as zoster. Interestingly, many of the zoster cases were clinically misinterpreted as HSV, suggesting that this diagnosis is frequently missed in clinical practice and could potentially be underdiagnosed [8].

Because of the potential misdiagnosis, it's key to remember illness scripts for HZ. The risk is greatest for those over 50 years old, with the median age of onset being 56 years old [9]. Although immunocompromised patients are at increased risk, more than 90% of HZ cases occur in immunocompetent individuals, as reactivation of latent VZV can still occur in the setting of age-related decline in cell-mediated immunity or other transient alterations in immune surveillance [8]. Initial presentations are similar to herpes simplex, with a pre-eruptive stage involving burning and pain at the site of the future rash, followed by the eruptive phase with vesicles or ulcerations. A potentially differentiating feature during the eruptive phase is the dermatomal distribution and unilateral presentation.

A definitive diagnosis of genital VZV versus HSV is crucial because the two infections are managed differently. For instance, genital HSV is treated with 1 gram valacyclovir twice per day for seven to 10 days, whereas VZV is treated ideally within 72 hours of the outbreak with 1 gram of valacyclovir three times per day for seven to 10 days. In addition to differences in valacyclovir or acyclovir dosing for treatment, VZV has a lower chance of recurrence compared to HSV and is not classified as a sexually transmitted infection [9]. This distinction influences recommendations regarding sexual activity and the management of discordant partners. Patients diagnosed with HSV are encouraged to disclose their diagnosis to sexual partners, as this communication has been shown to reduce transmission rates [10]. In contrast, such discussions are not indicated for individuals with HZ, which is not sexually transmitted. In cases of serodiscordant couples, suppressive therapy with valacyclovir is recommended as an additional strategy to decrease the risk of transmission and is specifically indicated for HSV-infected individuals [11,12]. If suppressive therapy is being considered, the partner’s HSV serostatus should be assessed through serologic testing to confirm lack of prior exposure, and if negative, suppressive therapy in the infected partner is warranted. Thus, an HSV diagnosis often necessitates additional counseling, testing, and medical interventions that are not required in cases of HZ. Subjecting a patient to these steps unnecessarily may result in undue emotional and psychological burden.

Accurately distinguishing between HSV and HZ not only guides appropriate medical management but also helps to avoid potentially serious social and relational consequences. A potential HSV diagnosis can lead to relationship strain or distrust, particularly when patients or partners presume that transmission must have occurred recently or through infidelity. While a new presentation of HSV does not definitively indicate acquisition from the current partner, the social implications can be significant. Furthermore, HSV infections carry a significant stigma, often leading to feelings of shame, anxiety, and depression, despite their widespread prevalence [13-15]. A systematic review of 30 studies on individuals with primary or recurrent genital herpes found that the infection can contribute to depression, lower self-esteem, stress, anxiety, isolation, and negatively impact work, school, and sexual relationships. By providing a definitive diagnosis that rules out HSV, clinicians can help patients avoid the mental health burden associated with an assumed HSV diagnosis.

Recent large-scale population-based studies further highlight the ongoing clinical burden of HZ, with incidence estimates consistently ranging from approximately three to 10 cases per 1,000 person-years in both US and European populations, underscoring its continued relevance in contemporary clinical practice [16,17]. In light of this, a practical diagnostic approach is to perform HSV PCR as the initial test in most genital vesicular eruptions, while adding VZV PCR when lesions are unilateral, dermatomal, and associated with neuropathic pain, or when HSV testing is negative despite a high clinical suspicion.

## Conclusions

In conclusion, genital zoster, though uncommon, presents a diagnostic challenge due to its similarity to the more prevalent HSV infections. A definitive diagnosis is important for appropriate management, as VZV requires different treatment and has a lower risk of recurrence compared to HSV. The stigma surrounding HSV infections further underscores the importance of accurate diagnosis, as it can have significant mental health implications for patients. Clinicians may consider testing patients with new-onset genital vesicles or ulcers for both HSV and VZV or maintaining a high index of suspicion in cases with suggestive clinical features, such as a dermatomal distribution or in immunocompromised individuals. By improving awareness and diagnostic accuracy, healthcare providers may better address both the clinical and psychological needs of patients.
